# Different aspects of therapy limitation: a comparative study of the nurse's view

**DOI:** 10.1186/cc12465

**Published:** 2013-03-19

**Authors:** O Szücs, G Élö, L Szabó, C Varga, J Gál, L Zubek

**Affiliations:** 1 Semmelweis University, Budapest, Hungary; 2 University of Kaposvár, Hungary

## Introduction

During the last few years the frequency of end-of-life decisions (EOLD) significantly increased in ICUs. The method of nurse involvement in making EOLD is different worldwide [1,2]. The purpose of this study was to analyze opinions of nurses about therapy restriction. We have examined with a multicenter study the opinions of the medical stuff about end-of-life care in Hungarian ICUs.

## Methods

We performed a questionnaire evaluation among physicians and nurses of ICUs about influencing factors of therapy restriction, the method of the decision-making process, and the frequency of different EOLD. The questionnaire, containing 21 questions, was delivered electronically to Hungarian ICUs, and then we analyzed the responses anonymously. The retrieved 302 answers (191 physicians, 102 nurses) were analysed using a nonparametric Student's test.

## Results

A total 71% of the nurse responders work in university clinics, 2% in regional centrum, 24% in municipal hospital, 3% in other ICUs. The nurses found both human (2.72/5 vs. 1.98/5) and material (2.81/5 vs. 2.12/5) resources more restrictive factors during patient admission than physicians (*P *= 0.025, *P *= 0.0024). Nurses working in municipal hospital were more strongly influenced by lack of material and human resources (3.34/5, 3.3/5) than nurses working in university clinics (2.2/5, 2.43/5), *P *= 0.01, *P *= 0.025. Younger nurses (working between 6 and 10 years) were more interested in the patient's or surrogate's wishes than older nurses (working more than 10 years). Religion did not influence patient admission and forego therapy; however, religious nurses compared with atheists and nonpracticing believers preferred to prolong therapy against the patient's will (*P *= 0.04). Nurses felt that physicians slightly involved them in the end-of-life decision-making process (2.1/5 vs. 2.4/5 *P *= 0.0001).

## Conclusion

We found that the workplace, level of medical attendance, godliness, work experience, and position in medical staffstrongly influenced making EOLD. While limitation of the therapy should be team work, nurses felt their opinions were hardly taken into consideration, although nurses seemed to be more realistic in the decision-making process.
